# Equity in access to fortified maize flour and corn meal

**DOI:** 10.1111/nyas.12306

**Published:** 2013-12-11

**Authors:** Gerardo Zamora, Luz Maria De-Regil

**Affiliations:** 1 Universidad Pública de NavarraPamplona, Spain; 2 Department of Nutrition for Health and Development, World Health OrganizationGeneva, Switzerland

**Keywords:** fortified maize flour, social determinants of health, equity, accessibility, fortified corn meal

## Abstract

Mass fortification of maize flour and corn meal with a single or multiple micronutrients is a public health intervention that aims to improve vitamin and mineral intake, micronutrient nutritional status, health, and development of the general population. Micronutrient malnutrition is unevenly distributed among population groups and is importantly determined by social factors, such as living conditions, socioeconomic position, gender, cultural norms, health systems, and the socioeconomic and political context in which people access food. Efforts trying to make fortified foods accessible to the population groups that most need them require acknowledgment of the role of these determinants. Using a perspective of social determinants of health, this article presents a conceptual framework to approach equity in access to fortified maize flour and corn meal, and provides nonexhaustive examples that illustrate the different levels included in the framework. Key monitoring areas and issues to consider in order to expand and guarantee a more equitable access to maize flour and corn meal are described.

## Introduction

Micronutrient malnutrition (MNM) affects all regions of the world. Countries in every region face deficiencies in individual or multiple vitamins and minerals (iron, folic acid, vitamin A, vitamin B12, or zinc). It is estimated that micronutrient deficiencies may currently affect one-third of the world's population.^1^ The consequences of MNM are generally well documented.^2^,^3^ It has been calculated that 53 million disability-adjusted life years and 1.5 million deaths of children who are under 5 years of age are linked to MNM.^4^ The negative impact of MNM on people's productivity, opportunities, and health outcomes make them more vulnerable to impoverishment.^5^ MNM is also associated with morbidity and mortality, and hinders improvements in maternal health.^6^–^11^

Fortification of foods with vitamins and minerals as a public health intervention aims to increase the micronutrient content in staple foods or condiments at the processing stage, before they are introduced to the market, as a means to improve the nutritional quality of the population's diet.^12^ Targeted fortification is commonly directed to specific subpopulations, for example, fortified complementary foods to be consumed by infants and young children^13^ or supplementary food for people living in emergency settings.^14^ Mass fortification, often market driven, involves fortifying staple foods that are consumed by a large sector of the population. Mass fortification can be voluntary or mandatory; in the former, food manufacturers decide to fortify their product for business reasons (e.g., ready-to-eat breakfast cereals), while mandatory mass fortification is a public health intervention enforced by a government to ensure the population receives adequate amounts of vitamins and minerals.^12^,^15^ Mass fortification of staple foods is a longstanding public health intervention that plays an important role in the effective and timely intention of the United Nations Millennium Development Goals (MDGs).^16^,^17^ Wheat flour fortification with iron or salt iodization, for example, has proved to be an effective intervention with a high effectiveness-to-cost ratio.^18^

More than 200 million people rely on maize, in any of its forms, as a staple food,^19^ especially in Sub-Saharan Africa, Southeast Asia, and Latin America. Estimates suggest that maize provides approximately 20% of the calories consumed in the world.^20^ In countries where maize is a staple, corn flour or maize meal tends to be consumed by population groups across the social gradient, irrespective of age, sex, socioeconomic position, or place of resident. However, populations in a lower socioeconomic position and living in less urbanized areas are more likely to have a heavy reliance on maize (flour or meal) as a dietary staple.^21^–^24^ By 2013, 12 countries had a policy to fortify maize flour with at least one micronutrient: five countries in Africa (Kenya, Nigeria, South Africa, Tanzania, and Uganda) and seven in the Americas (Brazil, Costa Rica, El Salvador, Guatemala, Mexico, United States of America, and Venezuela).^25^

Fortified foods do not always reach the population groups most in need of this intervention,^26^ and maize flour or corn meal is no exception. Inequity in access to fortified foods needs to be locally researched and contextually understood, just as food fortification needs to be understood and analyzed in country-specific contexts.^27^ The global public health community agrees that such inequity is socially determined and must be analyzed through a perspective of social determinants of health (SDH).^28^,^29^ This article on equity in access to fortified maize flour and corn meal draws on such a perspective, as well as on well-established analytical public health models, such as the World Health Organization (WHO) framework of analysis to approach equity, social determinants, and public health,^30^ which has been used to better understand other nutritional problems, such as the lack of consumption of fruits and vegetables in Chile^31^ and child malnutrition in Iran.^32^ Some implications of this analysis for program monitoring and policy are also presented. In this article, maize flour and corn meal are used as a generic term that comprises various types of maize flour and corn meals produced and consumed in different countries or regions of the world (e.g., nixtamalized or precooked flour).

## SDH and fortified maize flour and corn meal

SDH can be broadly defined as the conditions in which people are born, grow, live, work, and age.^33^ The WHO Commission on Social Determinants of Health (CSDH) laid out the conceptual foundations for the analysis of SDH and the relationship between health and other sectors.^34^–^36^ Based on a social production-of-health approach, this framework analyzes “individual health outcomes and diseases and their unequal distribution across population groups [which] are the result of the interaction of several determinants operating at different domains.”^36^

SDH explains how social, economic, and political mechanisms produce socioeconomic positions that stratify populations and individuals. Examples of stratifiers are place of residence, race or ethnicity, occupation, gender, religion, education, socioeconomic position, and social capital, also known by the acronym PROGRESS.^37^ These stratifiers are widely used for reviews in public health^38^ and are aligned with the recommendations of the WHO CSDH.^33^ They reflect people's positions within social hierarchies and act together with other structural and intermediary determinants that account for inequities in access to public health interventions. The social positions of individuals explain to a large degree the causes of their micronutrient deficiencies—a condition that, in turn, reinforces their social position, as MNM hinders individuals’ development and well-being. An SDH approach is helpful to assess whether fortification of maize flour and corn meal is an effective response to the needs of individuals across the whole social gradient, and whether it especially responds to the needs of the most vulnerable.

**Figure 1:**
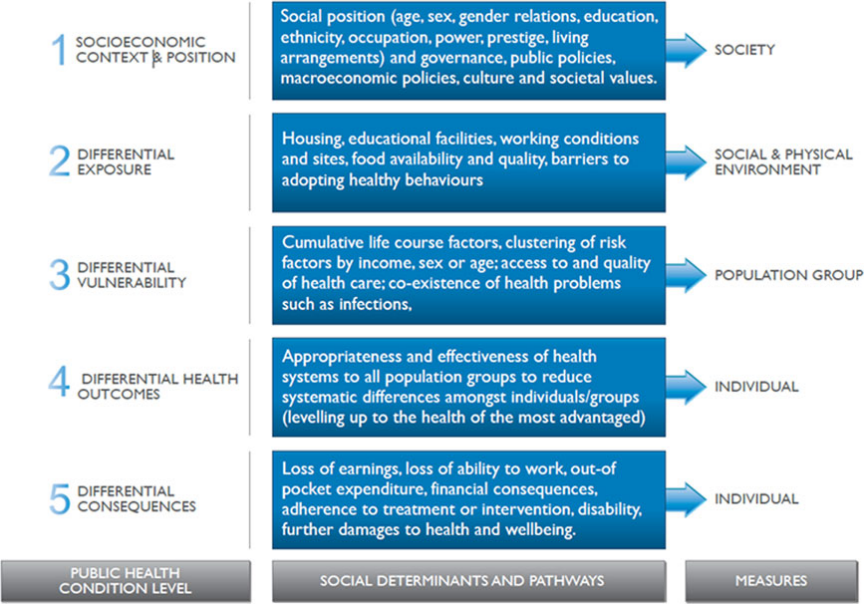
World Health Organization (WHO) priority public health condition analytical framework for understanding inequities in access to fortified maize flour and corn meal. Adapted, with permission, from Blas and Sivasankra Kurup.^30^

Access to fortified maize flour and corn meal is socially determined, as it is the result of several determinants operating at different domains (Fig. 1); similarly, the distribution of such access across the social gradient is also socially determined. The reasons why some population groups are more or less likely to access fortified maize flour and corn meal, and why inequities in access persist, are varied and often not well documented. Research on other types of fortified flour, such as wheat flour, suggests that in some settings, fortification is unlikely to benefit the neediest.^26^ Determining whether this is also the case for fortified maize flour first requires identification of the barriers that prevent equitable access in different fortification contexts. The following section proposes a framework to identify these barriers.

## Equity in access to fortified maize flour and corn meal

The concept of equity in health implies a need to address differences in health status that are judged to be unnecessary, avoidable, and unfair.^39^,^40^ These unjust differences are socially determined. In this view, health is a social phenomenon and health equity is understood as the absence of unfair and avoidable or remediable differences in health among social groups. Ideally, health equity implies that all individuals should attain their full health potential.

WHO has developed a five-level framework of analysis to approach equity, social determinants, and public health programs.^30^ This framework is suitable to approach MNM and equity in access to fortified maize flour and corn meal (Fig. 1). Like any framework, it is a simplification of a more complex reality and, indeed, some of its categories might be placed in more than one box. Yet it is a tool for “organizing the work from analysis to action in a manner that is consistent with the conceptual framework of the Commission on Social Determinants of Health.”^30^,^33^

Equity analysis in health is rooted in a larger equity analysis in development and human rights. Therefore, major international and intergovernmental organizations working in development have recently started to introduce an equity approach in their strategies and programs. For example, the United Nations Children's Fund (UNICEF) has an equity approach that seeks to identify, understand, and address the “causes of the causes,” that is, the roots of inequity in access to education, healthcare services, food, clean water, or legal protection that impede children's survival, growth, and development.^41^ Other agencies are using equity frameworks to approach health issues in development, most of which are based on a human rights and health perspective.^42^–^45^

The following sections underscore key issues around the five dimensions encompassed in the framework shown in Figure 1.^30^ Although not exhaustive, each section discusses the role of SDH and, whenever possible, the related inequities in terms of the three main measures of equity: (1) health disadvantages, owing to differences between population groups or between societies; (2) health gaps; that is, the differences between those that are the worst off and the rest; and (3) health gradients, pertaining to differences across all groups in the population.^33^,^46^

### Socioeconomic context and position

Socioeconomic status is a major determinant of nutritional status and food availability. At the country level, in general, low-income states have a higher prevalence of anemia and vitamin and mineral deficiencies than those with higher income.^47^ This association between income and anemia is also evident in high-income countries, where people of low socioeconomic status are especially susceptible to deficiencies in iron and other vitamins and minerals.^48^ Low-income families are more likely to base their diet on staple foods.^49^–^51^ Therefore, access to fortified maize flour and corn meal seems to be mediated by their costs.

#### Income, cost of food, and purchasing power of families

Although it is possible that households in rural areas are sometimes able to grow and produce some of the foods they consume, evidence suggests that cost is a key determining factor that influences the nature and amount of foods consumed, including fortified foods. This is a consistent observation in studies carried out in different countries and settings.^26^,^51^–^56^

Access to fortified foods may also be hindered by the low purchasing power of the individuals who usually need the intervention.^57^–^59^ Moreover, commercially fortified maize flour and corn meal can be available, but not accessible, owing to slightly increased costs, especially when fortification is voluntary.^52^ In some countries, such as Guatemala, rural and low-income households are more likely to purchase tortillas or other maize-based foods in local markets rather than purchase industrially processed foods, or to grow their own crop of maize and grind or mill them locally to produce corn masa or flour.^4^ This might explain why in countries such as Guatemala, where maize is a staple, household income and expenditure surveys find so little consumption of maize flour.^4^

Increases in the cost of food affect the micronutrient status of the population. In 2002, it was suggested that the escalation of the cost of the common diet in Venezuela was one of the leading causes of the increase in micronutrient deficiencies.^56^

#### Education level, attitudes, knowledge, and misconceptions

Consumers’ preferences (and thus potential purchases) seem to be modifiable if the consumers have some understanding of the benefits of consuming fortified maize products. A study carried out in Kenya, using an experimental auctions methodology, found that participating consumers were willing to pay substantially more for fortified maize, which had an average premium of 24.6% over nonfortified maize.^51^ Even though subjects participating in the study possessed limited knowledge on nutritional quality, most were aware of the existence of fortified maize and of night blindness caused by vitamin A deficiency. In this case, willingness to pay seems to be mediated by knowledge of the consequences of vitamin A deficiency and the need to avoid it. In general, evidence suggests that fortified foods might be principally consumed by more educated groups or by those with more stable income—groups that are not always at greatest risk of micronutrient deficiencies.^53^

Several studies and pilot projects carried out in various African countries have identified misconceptions about food fortification. After qualitatively interviewing adult men and women in Malawi for a World Vision project on small-scale hammer mills, several barriers to acceptance that were impeding access to fortified maize flour were reported.^60^ The most pervasive of these misconceptions was the idea that the blends used to fortify maize flour contained poison or contraceptives as part of a plot to limit family size. This finding suggests that other social processes and projects (in this case, family planning) might have been misled or misunderstood and, unexpectedly, might be influencing people's acceptability of another foreign project, such as food fortification, which is usually carried out with the cooperation of international aid. Moreover, these findings highlight the importance of understanding and respecting cultural values, such as those around the family (see the section Differential vulnerability), and also the need to train local mill operators, which can act as local disseminators of true knowledge. Misconceptions, too, can lead to fortification being attributed to some unlikely and false benefits, such as improving sexual strength or directly increasing birth rates, as was also found in this same study.^60^ Even though such ideas may serve as encouragement to consume fortified maize flour, it is unethical not to prevent this misbelief from spreading. In another study, data were analyzed from 2619 postpartum women in Honduras^61^ and showed that, despite the fact that 88.5% received some sort of prenatal care, almost one-quarter of the women (23%) believed congenital anomalies are related to a superstitious or mythical cause, and only 18.1% mentioned lack of vitamins or micronutrients as a possible cause. This lack of appropriate knowledge about the role of food intake during pregnancy may play a role in an individual's consumption of fortified maize flour and corn meal.

#### Cultural norms, gender roles, values, and intrahousehold distribution of food

Households are the loci for the expression of cultural values related to food.^62^ The distribution patterns of food allocation based on differential valuation of household members may include, for instance, the favoring of men over women, adults over children, or vice versa. Differential allocation is more likely to operate for those foods that are perceived as luxury foods, or when food availability is scarce. Findings suggest that if differential food allocation occurs, it rarely is applied to staple foods.^53^,^54^,^63^–^65^ Whether food allocation behaviors operate with regard to fortified foods such as maize flour and corn meal is unknown; such a situation would most likely affect women, girls, and elderly individuals.

#### Support from local leaders

Local leaders can be instrumental in facilitating or blocking an intervention. In some African countries, for example, food fortification has been wrongly associated with family planning. These misconceptions may block access to fortified maize flour and corn meal, especially if they are supported by local leaders or traditional authorities. Research on a pilot project in Malawi found that once these leaders or authorities understood what fortified foods are and how they work, they campaigned to get more small-scale mills in their towns and villages and were indeed raising awareness on the importance of fortifying maize flour.^60^,^66^ These allies need to be involved in public health strategies to help build political will.

#### Policies and regulatory frameworks

Mass fortification of maize flour and corn meal has a long way to go. Currently, more than 70 countries have legislation to mandate wheat flour fortification, while only 12 mandate maize flour or corn meal fortification.^25^ Information from Morocco, Uzbekistan, and Vietnam shows that the existence of legislation and standards for mandatory fortification is key to improving the reach and success of fortification programs.^67^

While usually being a national-level intervention, food fortification is highly entrenched in the politics of global food regulations, whose political agenda may be constructed by a complex interaction of public and private interests. Legislation is necessary for establishing a fortification infrastructure and sanctions for noncompliance, as well as for marketing the products so that they address the consumers’ demand.^67^,^68^ Legislation is also the way to level the business playing field and protect those millers who would be supportive of fortification but would not be able to recover their investment and costs from the market, if being at a disadvantage.

However, in some countries or contexts, there may be resistance to mandatory fortification of flour, and this may hinder the progressive use of fortified maize flour or corn meal. Inadequate marketing strategies and concerns from human rights advocates^69^,^70^ or some stakeholders worried about the potential adverse consequences of food fortification^71^ can generate difficulties for the promotion of fortified maize flour and corn meal. Evidence-informed social marketing, which targets information to promote consumer awareness of products among the groups that most need them, may be a useful tool to overcome this barrier. For instance, evidence from Côte d'Ivoire and Kenya suggests that social marketing builds demand and increases consumption of fortified foods, including fortified maize products for children.^72^ Furthermore, the United Nations Special Rapporteur on the right to food, has called global actors to ensure that food policies and initiatives, including fortified foods, and especially those involving public–private partnerships, observe human rights standards.^73^

#### International trade policies

Trade agreements may also challenge the implementation of maize flour and corn meal fortification. Any country considering fortification should address its international and regional trade obligations. In general, the World Trade Organization (WTO) requires nondiscrimination between partners that have signed treaties as well as nondiscrimination between imported and locally produced goods. Exceptions to the WTO principles of nondiscrimination allow member states to adopt trade measures “necessary to protect human, animal or plant life or health” if they prove that such measures are necessary to meet a public health need and that they are not a disguised attempt to restrict trade or promote discrimination. A country that is considering maize flour or corn meal fortification as part of their public health programs needs evidence-informed standards compliant with those established by normative organizations. Any trade regulation waiver would consider a public health problem and would ensure that the same requirements are imposed on local and imported products.^74^

### Differential exposure

Individuals and population groups at higher risk of MNM are usually also at higher risk of many other social and health problems. Public health programs must differentiate these risks across the various social gradients and adjust interventions accordingly.

#### Distance and hard-to-reach fortification mills

Distance can be a major barrier to accessing fortified maize flour and corn meal. A study in Malawi that included provision of fortified foods to children and their families found that travel and distance may be major barriers to adhering to the intervention.^75^ Similarly, time-consuming activities linked to these interventions, such as training and talks, may become barriers too.^54^,^75^ From the millers’ perspective, a study in Zimbabwe found that distance plays a major role as the “the further the premix has to travel to get to the points of supply to the hammer mills, the higher the cost of freight per unit of maize fortified at the hammer mills.”^76^ These costs may have a negative impact on the affordability of fortified maize flour and corn meal.

#### Food availability, changes in consumption patterns, and rural/urban differences

Changes in food availability and changes in consumption patterns are likely to affect consumption and access to maize flour and corn meal. In Venezuela, the increasing availability of wheat flour, and of products based on wheat flour, was cited as a potential reason for decreases in consumption of precooked maize flour.^77^ Similarly, differences between South African school-age children in urban and rural areas highlighted that those in rural contexts were more likely to consume maize porridge, and in larger quantities, than children in urban settings.^78^ Those in urban settings were found to consume much more white bread. Therefore, mandatory fortification of maize flour was more likely to have a greater impact on rural children's intake of micronutrients and minerals. Moreover, these findings support the suggestion that having accurate and available data on household consumption patterns is key to successfully integrating fortification efforts into public health programs in low- and middle-income countries.^4^

#### Displaced populations and long-lasting deprivation

Populations facing longstanding deprivations and stressful conditions may face barriers to accessing and adopting fortified foods. Such is the case of displaced populations and refugees, who often face physical conditions that are likely to impede adequate access to micronutrients and minerals. Their situation is complex; they face a set of barriers to accessing not only fortified foods but also food in general, such as a lack of fresh food; they face poor livelihoods and limited access to markets.^79^

Seal *et al*. assessed changes in iron and vitamin A in the former Nangweshi refugee camp in Zambia, before and after the deployment of a multiagency project that provided custom mobile milling and fortification equipment to allow the production of fortified maize meal at the refugee camp.^80^ The project particularly sought to involve potential beneficiaries of the intervention as production staff. An association was found between the introduction of fortified maize meal and improvements in the iron and vitamin A status of camp residents, especially for adolescents and children. The key to the achieved improvements was largely based on the approach (custom mobile milling and fortification equipment, with local involvement). This approach could be adapted to other contexts and circumstances, in order to be replicable in other food aid programs.

### Differential vulnerability

Socioeconomic groups may be affected differently by the same factor or circumstance. Clustering of risk factors, or their cumulative effects throughout the life course, make underprivileged populations more vulnerable to facing barriers to accessing fortification of maize flour and corn meal.

#### Cultural values, body image, and family planning

Mandatory food fortification does not require any change in individual behavior, as it takes advantage of the regular diet. However, changes in the regular diet may be affected by several factors, including those related to age and gender,^81^ which are usually hard to address by large-scale interventions. A study in Brazil suggests, for example, that cultural values and norms related to women's expected patterns of beauty may play a role in the quantity and quality of food consumed by adolescents, especially when pregnant,^82^ which may be influencing their nutritional status. Similar findings highlight the importance of addressing the increase in (unhealthy) dieting habits of adolescents and young women because of patriarchal beauty expectations, in countries facing a nutritional transition.^83^ Similarly, the impact of fear and stigma in cultural contexts where adolescent pregnancy is both increasing and badly regarded may affect the diet.^60^ For example, adolescent girls and young women may hide their pregnancies in their first months. Hiding can imply eating less and generally little food, in order to stay thin. These behaviors affect the neonate's weight, but also the micronutrient intake of the pregnant women. Linkages between pregnancy, family planning, beliefs, behaviors, and fortified foods are complex and need further study (see the section Socioeconomic context and position).

#### Clustering of risk factors

A combination of limited opportunities for education, jobs, and income generation affects access to fortified foods, and the populations that most need the intervention may be the ones with less access to them. Clustering of risk factors is increasingly being addressed through conditional transfer programs or food assistance schemes. A randomized effectiveness evaluation of the Oportunidades program in Mexico among rural children aged 12–59 months, found that the distribution of fortified foods had a positive effect on the nutritional status of these preschool children.^84^ However, other evaluations and comparisons of conditional transfers and food-based programs in Peru, Chile, Brazil, and Mexico suggest that schemes based only, or largely, on distribution of foods do not generate high social welfare gains, as observed in conditional cash transfer programs.^85^ Some studies have found that cash transfers work best and are most cost-effective in areas where markets function appropriately, while food assistance works best and is most cost-effective in areas where markets are less functional or accessible.^86^ Thus, mass fortification of maize flour and corn meal must take into account differences related to the market where the intervention takes place.

Public health interventions, such as distribution of fortified maize flour and corn meal, may be affected by these factors when the intervention is not market based. Even though fortified foods cannot be expected to reach all deficient populations, they can make a difference for the large and expanding populations of all socioeconomic classes that regularly purchase and consume commercially processed foods.^87^

### Differential health outcomes

Health systems work to level up unjust differences. For example, anemia, a condition that affects over 1.6 billion people worldwide,^2^ tends to affect specific groups disproportionately, according to sex, age, race/ethnicity, wealth, and place of residence. Some of these populations largely based their diets on maize products.

#### Health systems

Despite fortification of foods being a market-based intervention, it may benefit from a strong health system, which is a powerful determinant for health.^29^ The WHO Health Systems Framework includes seven building blocks: leadership/financing, healthcare financing, health workforce, medical products and technologies, information and research, and, finally, service delivery. Evidence is limited on the role of each specific building block in relation to access to fortified maize flour and corn meal.

Social participation approaches and the increased involvement of consumers of fortified maize flour and corn meal have enhanced access to these products, as suggested by evidence from Côte d'Ivoire and Kenya,^72^ as well as Malawi.^60^ The involvement of local leaders in the promotion of fortified maize products, as a means of respecting local cultural values, has also been shown to be a key determinant.^66^ No evidence has been found on health impact assessments, either mandatory or voluntary, concerning policies and programs for fortification of maize flour and corn meal.

### Differential consequences

Unexpected difficulties are likely to have unequal consequences for individuals and their families, as each situation and individual has different associated baseline conditions. However, individuals and populations in poor living conditions frequently have fewer resources to surmount unforeseen adversities. Social protection regimes seek to reduce households’ and individuals’ vulnerability to such unanticipated adversities and longstanding deprivations.^88^

#### Increasing costs of energy

Consumers of maize flour or corn meal largely use local mills to transform their corn into masa or flour. Fortification usually takes place at the mills, and many mills are fuel operated, while others are electrically powered. Local mills are more vulnerable to increases in fuel prices than are large industries producing commercially distributed maize flour.^5^,^76^ Slight increases in fuel price are rapidly translated into slight increases in the cost of milling and fortification. However, a small increase may not be insignificant for very poor households or individuals, who may be willing to forego the cost of fortification for reasons of affordability coupled with a lack of understanding about the benefits. Several studies in African countries, such as Zimbabwe,^68^ have found that escalating prices of diesel erodes the profitability of diesel-powered hammer mills. Since customers are sensitive to the cost of service milling (increases in milling fees), there have been cases of diesel hammer mills being shut down if there are electrically powered mills within walking distance.

Different schemes have been carried out to influence food choice. Pricing strategies (food taxes and subsidies) have been proposed as a means to improve population diets.^89^ This approach has been followed in Egypt, where wheat flour is fortified with iron and folic acid and the resulting baladi bread is sold at subsidized prices.^90^ Clearly this approach needs further study, as evidence is limited for maize flour and corn meal. Additionally, evidence on the impact of social protection regimes on promoting equitable access to fortified maize flour and corn meal is also scarce.

## Implications for monitoring and policy

Fortification of maize flour and corn meal requires intersectoral action for policy making, deploying interventions, and monitoring of its impact. As exemplified throughout this article, interventions need to take into account a wide range of social determinants. The conceptual framework used for this analysis offers a valuable tool to identify the lessons learned, potential entry points for interventions, and the sectors that have a role in increasing access to fortified maize flour and corn meal. Table 1 presents a summary of some preliminary suggestions following this direction. It does so in a limited manner, highlighting only those questions directly linked to fortification of maize flour and corn meal that may be subject to monitoring and, if necessary, intervention. The challenges faced by health systems in collecting and using the key information required to assess and address health inequities are well known.^91^,^92^ These challenges may be even bigger in countries where fortification is not governed by the health sector, but instead is administered by the trade ministry or the social protection ministry.

**Table 1:** Inequities in access to fortified maize flour and corn meal: suggested pathways, entry points, interventions, and measures

Public health			Potential adverse	
condition level:		Potential entry	side effects and	
pathways/	Interventions and lessons	points for	sources of	Sectoral
determinants	learned	interventions	resistance	responsibilities
**Socioeconomic context and position**	Enact laws guaranteeing better access to education, employment, adequate housing, and health, which are determinants of access to food. Design fortification policies that are culturally appropriate and acceptable. Enact policies that redistribute wealth and resources, especially income differences that might impede access to commercial fortified maize flour. Design policies and interventions aimed at changing values and norms that impede adequate nutrition, including access to fortified food. Improve women's access to education and health services. Identify existing knowledge of fortified foods, MNM, and related diseases, as a means to increase self-awareness. Identify household distribution patterns of food allocation.	National legislative bodies Education system Employers Management of healthcare facilities Food industry	Resistance of political lobbies Resistance from groups opposing redistribution policies Traditions and costumes regarding food intake and other social behaviors Resistance from the food industry to change production patterns Difficulties of cash transfer and other policy interventions to incorporate fortified maize flour	Not a health sector responsibility, but common shared objective across sectors
**2. Differential exposure**	Provide free fortified maize flour or corn meal to the groups that are more likely to consume them. Provide appropriate means for fortification, especially for village mills and other nonindustrial maize production sites. Guarantee that school meals include fortified maize bread/buns or porridge. Coordinate with the education sector, and other sectors working with youth, to address the differential exposure of pregnant adolescent girls to MNM in order to increase their access to fortified foods. Identify barriers related to distance and travel costs.	Community centers (civic and religious) School system Healthcare facilities Locations most frequently visited by adolescents	Traditions and costumes regarding food intake and other social behaviors Resistance from owners of village mills or community mills	Health sector in alliance and coordination with other sectors and private actors
**3. Differential vulnerability**	Improve early detection of micronutrient deficiencies in individuals and communities. Improve access to health promotion programs for the most vulnerable groups at risk of MNM. Combine poverty reduction strategies with incentives/mandates to use fortified maize flour and corn meal. Improve women's access to education and health services. Identify differences in school attendance of school-age children.	Healthcare facilities Social services facilities Community facilities Civic organizations and other socially organized groups Policies and programs aimed at women's empowerment	Resistance from nonpublic health sectors Resistance from different organizations Cultural resistance to empowerment of women and to combating discrimination against women	Health sector in alliance and coordination with other sectors and private actors
**4. Differential health outcomes**	Set up policies that aim at adherence to using fortified maize flour and corn meal (while continuing to carry out policies aimed at introducing fortified maize flour and corn meal). Increase awareness of public health and other public officers of pathways and determinants of inequity in access to fortified foods and other strategies against MNM. Set indicators to monitor the differential impact of policies on fortification in order to design policy innovations that level up the most disadvantaged (e.g., health impact assessments).	Healthcare facilities Social services facilities Community facilities Civic organizations and other socially organized groups	Resistance from nonpublic health sectors Resistance from different organizations	Health sector in alliance and coordination with other sectors and private actors
**5. Differential consequences**	Fully integrate an equity-in-health perspective in public health interventions. Identify MNM consequences on life opportunities and strengthen public awareness of the role fortified foods (e.g., maize flour and corn meal) can have in tackling these inequities in opportunities. Appropriately inform the most vulnerable populations of the long-term beneficial effects of consuming fortified maize flour and corn meal.	Social protection systems and schemes	Resistance from public health and nonpublic health sectors on adopting a perspective of equity in health Traditions and costumes regarding food intake and other social behaviors	Health sector mainly responsible

## Conclusions

The evidence and examples presented in this article suggest that incorporating an SDH and equity approach can contribute to increasing and guaranteeing access to fortified maize flour and corn meal. However, this approach is not yet common.

Food fortification is a complex public health intervention, and so strategies for fortification need to be intersectorally aligned, especially with poverty reduction programs and other social intervention schemes. Although it has been suggested that the long-term sustainability of fortification programs can be ensured when consumers are willing and able to bear the additional cost of fortified foods, this is exceptionally difficult in contexts of extreme and extended poverty and lack of opportunities. Thus, guaranteeing access to fortified foods requires that the reasons for the causes be addressed, that is, the factors that allow for the reproduction of exclusion and poverty that are socially determined and therefore modifiable.

Inequities in access to fortified maize flour and corn meal, where these are staples, can perpetuate inequalities among communities and individuals with respect to cognitive abilities, work skills, or capacities for self-protecting one's health and that of one's family. For the 900 million people that consume maize and maize-based products as their main staple food, it is crucial that the scientists, program implementers, and policy makers understand and intervene in the barriers that prevent access to fortified maize flour and corn meal.
